# Bacterial Exopolysaccharides: Insight into Their Role in Plant Abiotic Stress Tolerance

**DOI:** 10.4014/jmb.2105.05009

**Published:** 2021-06-21

**Authors:** Neeta Bhagat, Meenu Raghav, Sonali Dubey, Namita Bedi

**Affiliations:** Amity Institute of Biotechnology, Amity University Uttar Pradesh, Sector 125, Noida 201301, India

**Keywords:** Exopolysaccharides (EPS), drought, abiotic stress, salinity, metal, biofilm

## Abstract

Various abiotic stressors like drought, salinity, temperature, and heavy metals are major environmental stresses that affect agricultural productivity and crop yields all over the world. Continuous changes in climatic conditions put selective pressure on the microbial ecosystem to produce exopolysaccharides. Apart from soil aggregation, exopolysaccharide (EPS) production also helps in increasing water permeability, nutrient uptake by roots, soil stability, soil fertility, plant biomass, chlorophyll content, root and shoot length, and surface area of leaves while also helping maintain metabolic and physiological activities during drought stress. EPS-producing microbes can impart salt tolerance to plants by binding to sodium ions in the soil and preventing these ions from reaching the stem, thereby decreasing sodium absorption from the soil and increasing nutrient uptake by the roots. Biofilm formation in high-salinity soils increases cell viability, enhances soil fertility, and promotes plant growth and development. The third environmental stressor is presence of heavy metals in the soil due to improper industrial waste disposal practices that are toxic for plants. EPS production by soil bacteria can result in the biomineralization of metal ions, thereby imparting metal stress tolerance to plants. Finally, high temperatures can also affect agricultural productivity by decreasing plant metabolism, seedling growth, and seed germination. The present review discusses the role of exopolysaccharide-producing plant growth-promoting bacteria in modulating plant growth and development in plants and alleviating extreme abiotic stress condition. The review suggests exploring the potential of EPS-producing bacteria for multiple abiotic stress management strategies.

## Introduction

Abiotic stresses like drought, salinity, heavy metals, and high or low temperature are major constraints to crop production by being detrimental to the physical, metabolic, and growth development of plants [1]. Plants are often subjected to multiple stresses which are aggravated by climatic changes, use of chemical fertilizers, pesticides and environmental pollution. This situation is more alarming with the increase in world population which is expected to reach around 10 billion by 2050 [2]. There is an urgent need to increase food production by 70% to meet the demand [2]. It is also imperative to find ways to implement new agricultural strategies to protect crops from these multiple abiotic stressors [3].

Currently, there are several approaches that enhance plant tolerance to abiotic stress. These include water-conserving irrigation strategies, traditional methods of breeding, and genetic engineering of transgenic plants with abiotic stress tolerance [4]. Plant growth-promoting rhizosphere microorganisms are also now being widely used for restoring soil fertility, remediation of chemical pollutants and to sustain plant growth [5]. They are a proven, effective alternative to conventional methods and a promising strategy for mitigating abiotic stresses. The use of plant growth-promoting microorganisms is a simple alternative approach to genetic engineering and breeding methods for crop improvement since these procedures are time-consuming, expensive, and laborious [6]. These microorganisms improve the root and shoot growth, thus enhancing the water and nutrient absorption from soil [7]. Different types of plant metabolites, such as HCN, 2,4-diacetylphloroglucinol (DAPG) [8], antibiotics, *e.g.*, phenazine [9], and volatile compounds [10] help to enhance plant growth [11-13]. Exopolysaccharides, are produced by an array of microorganisms like bacteria, cyanobacteria, microalgae, yeasts, and fungi [14]. These exopolysaccharides impart defense against a wide range of environmental stresses like drought [15], metals [16], salt [17], and temperature [18]. Additionally, EPS facilitate microbe-microbe and microbe-plant interaction, provide antioxidants, store carbon, and supply nutrients to support plant growth [6, 19]. Diverse bacterial species like *Pseudomonas aeruginosa*, *Azotobacter vinelandii*, *Sphingomonas paucimobilis*, *Azotobacter*, *Paenibacillus*, *Klebsiella*, *Bacillus*, and *Pseudomonas* spp. produce EPS and play an important role in sustaining plant growth in abiotic stress environments [20]. The root microbiome, specifically rhizospheric microbes produce phytohormones, 1-aminociclopropane-1-carboxylase (ACC) deaminase, and EPS to sustain plant growth in drought. Crosstalk between phytohormones such as auxins, ethylene, gibberellins, cytokinins, jasmonic acid and transcription factors play a significant role in this abiotic stress tolerance [21].

Interestingly, the composition and concentration of the EPS that is produced varies in different strains, leading to various structural and functional types of EPS [22].The quantity and components of the EPS produced by bacteria depend on several factors such as type of bacteria, time of cultivation, state of growth, and availability of substrate [3, 23]. Therefore, the diversity of constituents present in the EPS imparts unique properties such as enhancement of water absorption and holding capacity of bacteria in the soil by means of aggregate formation [24]. Although EPS-producing bacteria are ubiquitously distributed in nature, there is a gap in knowledge with regard to the factors that are involved in the regulation, synthesis and variations in composition of EPS produced by plant growth-promoting bacteria in response to different abiotic stresses.

EPS-producing strains are more efficient in imparting resistance to abiotic stress compared to plant growth-promoting rhizosphere bacteria originated from the non-stress ecosystem [25]. Presence of these EPS strains influences plant growth and thus such bacteria are promising candidate biostimulants for stress management strategies for plant growth and development [25]. In the present review we discuss the role of exopolysaccharide-producing bacteria in combating abiotic stress in plants.

## Bacterial EPSs

EPSs are extracellular polymers of bacterial origin. They are produced in response to environmental signals as an energy exchange mechanism [26]. ESPs are a complex mixture of diverse types of biomolecules forming a 3D structural matrixome containing polysaccharides, sugars, structural proteins, enzymes, amino sugars, nucleic acids, lipids, pyruvates, glycoproteins, lipids, extracellular DNA, and some humic substances [23]. Each of these components vary in physicochemical properties and functions. Acetate esters, pyruvates, formates, and succinates are the common extracellular carbohydrate substituents found in EPS [27]. The EPS backbone contains repeating units of monosaccharides, classified as homopolysaccharides and heteropolysaccharides [28]. Homopolymers are made up of single sugars such as pentoses (arabinose, and xylose), hexoses (glucose, galactose, mannose, allose), desoxyhexoses (rhamnose, fucose), amino sugars (glucosamine and galactosamine), or uronic acids (glucuronic acids and galacturonic acids) linked by strong 1,4-*β*- or 1,3-*β*-l and more flexible 1,2-*α*- or 1,6-*α*-linkages. Heteropolysaccharides contain two to three different sugar moieties in repeated fashion. The carbohydrates may be linked to proteins (glycoproteins), lipids (glycolipids), acids (*e.g.*, glucuronic acid, galacturonic acid, or mannuronic acid), and/or extracellular DNA. The succinate and pyruvate substituents, uronic acids, and phosphate and sulfate residues, provide anionic charge to the matrix which easily captures many cations like metals, sodium, and potassium [29].

**Fig. 1:**
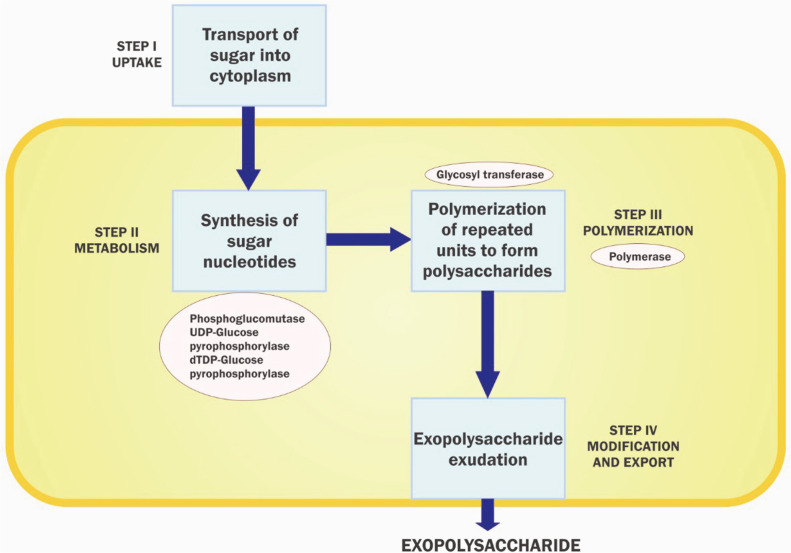
Steps involved in biosynthesis of bacterial exopolysaccharides.

These polysaccharides are responsible for cell adhesion, cell aggregation and water retention while forming a protective barrier and providing nutrient sources. In addition, proteins in the matrix provide binding enzymes (adhesins), electron acceptors and donors, and facilitate the absorption of organic and inorganic material. EPS amino acids serve as carbon and nitrogen sources for bacteria [30]. Nucleic acid also serves as source nutrients and genetic information. Humic substance supports adhesion and provides electron donor and acceptor [31]. In general, bacteria produce two types of EPS:capsular EPS (CPS), which is attached to the cell surface, and released polysaccharides (RPS-EPS), which are released by the bacterial cell into the surroundings. Extracellular enzymes of EPS facilitate nutrient absorption from aqueous medium and help in distribution as EPS changes from soluble to filamentous matrix form [32]. EPS helps in cohesion of bacteria, adhesion of biofilm to the root surface, and enables exchange of metabolic signaling information amongst bacteria to form a stable population with synergistic relationship [33]. There are 4 steps involved in the biosynthesis of EPS: 1) absorption, activation and conversion of monosaccharides into sugar nucleotides in the cytoplasm; 2) assembly of sugar nucleotide repeat units by the sequential addition of each unit on a lipid carrier molecule with the help of glycosyltransferase; 3) polymerization of these repeat units that occurs at the periplasmic side of the plasma membrane, and 4) exudation of EPS to the cell surface [32] (Fig. 1). The matrix of EPSs holds an assembly of microbial colonies to form biofilm. Formation of biofilms is regulated by multiple factors including metabolic signaling of quorum sensing [33]. Roots usually release secondary metabolite like phenolics, organic acids, amino acids and phytohormones, and siderophores in the form of root exudates. These exudates serve as chemoattractants for microbes associated with roots by electrostatic interaction and later develop the biofilm [34]. The quorum sensing (QS) system plays an important role in plant-microbe and microbe-microbe interactions in root harboring bacteria which promote plant growth during stress [35]. Strigolactones have been the signal molecules for plant microbe communication facilitating entry of bacteria into the roots of the plant. In bacteria two QS pathways (autoinducer-1 [AI-1] and autoinducer-2 [AI-2]) have been reported in bacteria (Miller, Waters) [35-37]. QS signal molecules, N-acyl homoserine lactones (AHLs), are commonly secreted in gram-negative bacteria, while autoinducer peptides are found in gram-positive bacteria. The AHLs serve as signaling molecules responsible for regulating the microbial population density in biofilm [36, 41]. The bacteria QS system helps bacteria acquire nutrition, ferric ions by siderophores, provides stress tolerance and produces antibiotics to inhibit pathogens [38]. QS signals also enhance root elongation, nodulation, and production of growth promoters like auxin and flavonoids to promote plant growth [38-40].

Microbial EPSs improve the quality and fertility of soil [41]. High-molecular-weight polymers in EPS help soil bacteria attach to the exterior of soil particles and sustain the collected particles. The EPSs adhere to soil surfaces through hydrogen binding, cation bonds, anion adsorption, and van der Waal’s forces [42]. By decreasing wetting and swelling, this organic outcome may facilitate the firmness of the soil aggregate aeration, permeation, root penetration, and decrease runoff [42]. EPS helps in microaggregation of soil by forming a organo-mineral sheath which improves the soil structure and provides stability to soil under stress conditions [41, 43] (Table 1).

**Fig. 2:**
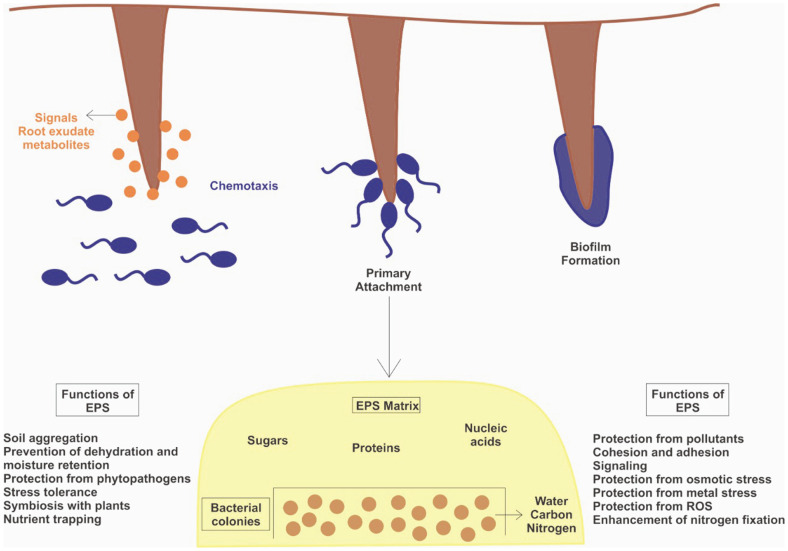
Roles of EPS microbial exopolysaccharides in mitigation of abiotic stress and plant growth promotion.

Many plant growth-promoting rhizosphere bacteria maintain plant-water relations, ion homeostasis and photosynthetic efficiency in plants under drought and salt stress. Stress alleviation involves a complex signalling network operating as a result of plant–microbe interaction [35]. Thus, EPS as nutrient facilitator and adhesive matrix for attaching microbes to plants plays an important mediator in maintaining plant heath in abiotic stress. A positive correlation exists between abiotic stress and production of EPS by bacterial cells [44] (Fig. 2).

## Role of EPS in Mitigation of Drought Stress in Plants

One of the major agronomic complications that affect crop yields in arid and semi-arid regions all over the world is drought stress [45]. Prolonged periods of drought up to many years and intensely acute environments of drought have the potential to lead to food scarcity in some countries [2, 46]. Drought-inflicted areas are considered high stress environments due to inaccessibility of water and variations in environmental humidity. The development and yield of crops are highly restricted in arid and semi-arid regions that are affected by drought stress and the properties and stability of soils are adversely affected [47]. As a result, it is essential to understand the effects of drought on other environmental phenomena such as soil aggregation in order to learn how plant growth can be enhanced under drought conditions [47].

Arid and semi-arid areas harbor different clusters of microorganisms that aid in the growth of plants in stressed environments by several mechanisms such as ACC, phytohormone regulation, antioxidant production, volatile organic compounds, and EPS production [13, 19].Various studies have been done to prove the important roles of EPS in inducing survival mechanisms under drought stress [2, 19]. As EPS is hygroscopic in nature, it helps in maintaining high water content for the survival of microorganisms under drought conditions [6].

As discussed previously, the EPS produced by different bacterial strains improves soil aggregation and increases water permeability and nutrient uptake around the roots, thereby supporting plant growth and protecting plants from drought stress [7, 12]. EPS produced by *Rhizobium* KYGT207 strain is rich in glucose (Glc), galactose (Gal), and mannuronic acid (ManA) and increases root-adhering soil (RAS) which facilitates the nutrient and water uptake in Tritium significantly [48]. The capsule of *Azospirillum brasilense* Sp245 strain was shown to possess complexes of high-molecular-weight components, such as complexes between lipopolysaccharides and proteins, and polysaccharides and lipids, which could offer protection under extreme environmental conditions such as desiccation [49]. Even upon decapsulation, cells of the *A. brasilense* Sp245 strain could survive under drought conditions [49]. *Pseudomonas mendocina* increased stabilization as well as soil fertility under drought stress [50]. Similarly, the EPS produced by *Pseudomonas putida* GAP-P45 strain reported to form biofilms on the surface of roots of sunflower seedlings, which promoted soil aggregate formation and enhanced stability of the rhizosphere region [19, 52]. This strain colonizes the rhizosphere and forms stable soil aggregates, increases plant biomass, and enhances the survival rate and RAS/root tissue (RT) ratio which promotes water and nutrient uptake thereby boosting plant survival under drought [52]. Under water deficit stress, EPS production was increased in different *Bacillus* species such as *B. licheniformis* strain HYTAPB18, *B. amyloliquefaciens* strain HYD-B17, and *B. subtilis* strain RMPB. Under drought stress HYTAPB18 and RMPB44 contained glucose and HYD-B17 produced raffinose as major sugar in EPS [53]. EPS, being fibrillar by nature, entangles with clay particles and forms microaggregates (<250 μm diameter) and macroaggregates (>250 μm diameter) by creating an intermediate zone where EPS and clay particles combine and become surrounded with bulk soil [19, 52].

When EPS-producing *Rhizobium* species were used as bioinoculants, they increased soil stability under drought conditions [54]. Similarly, *Microbacterium arborescens* has been shown to produce different polysaccharides that have the property of cementing soil particles together [55] (Table 1). Increase in levels of total carbohydrates in soils of arid regions is attributed to different types of EPS secreted by microorganisms [12, 34]. Maize seedlings that were primed with three different EPS-producing strains – *P. aeruginosa* Pa2, *Proteus penneri* Pp1, and *Alcaligenes faecalis* AF3 – demonstrated improvement in soil moisture, increase in plant biomass, root and shoot length, and surface area of leaves in maize plants under water deficit stress. The plants also showed an increase in their sugar, protein, proline, and relative water content, and a decrease in the activities of their antioxidant enzymes [56, 57]. Similarly, inoculation of wheat plant with EPS-producing *Mesorhizobium ciceri* CR-30 and CR-39, *Rhizobium leguminosarum* LR-30, and *Rhizobium phaseoli* MR-2 resulted in an improvement in plant growth and biomass and enhancement of the drought resistance capacity of wheat seedlings [58].

Drought stress increases levels of antioxidative enzymes and reactive oxygen species (ROS) in plants [59]. However, when these plants are treated with EPS-producing bacteria, they show a significant increase in the levels of catalase, ascorbate peroxidase, and glutathione peroxidase. *P. mendocina* showed the ability to augment catalase activity in lettuce under drought stress [51, 60]. *Rhizobium* species and *Serratia* species also enhanced drought resistance in lettuce by increasing antioxidant levels under drought stress conditions. Priming with these EPS-producing bacteria is reported to increase levels of antioxidant enzymes in several other plants like barley, wheat, sunflower, maize, and chickpea [11, 61].

High proline concentration supports maintenance of high turgor pressure, which is important for maintaining metabolic and physiological activities in plant cells under drought conditions [62]. Also, EPS-producing Plant Growth-Promoting Rhizobacteria (PGPR) such as *B. licheniformis* B642 and *Pseudomonas fluorescens* FAP2 strains were found to possess plant growth-promoting (PGP) traits such as production of siderophores, IAA, ammonia, and phosphate solubilization. Plants inoculated with these strains showed significant increase in vegetative growth, transpiration rate (E), chlorophyll content, stomatal conductance (g_s_), internal CO_2_ concentration (Ci), leaf water potential (LWP), and net photosynthetic rate (P_N_) as compared to uninoculated plants [62]. *Pseudomonas* species with EPS-producing ability have been shown to trigger the upregulation of the proline pathway to maintain high concentration of proline under drought stress [63]. Pepper plants treated with EPS-producing *B. licheniformis* K11 strain showed the presence of six differentially expressed stress proteins and a 1.5-fold increase in the products of specific genes such as vacuolar H^+^-ATPase (VA), dehydrin-like protein (Capsicum annuum) Cadhn, cytoplasmic small heat shock protein class I (sHsp), and Capsicum annuum pathogenesis-related protein 10 (CaPR) as compared to untreated plants exposed to drought stress [64]. EPS-producing *Planomicrobium chinense* and *Bacillus cereus* showed the ability to improve the sugar and protein content in leaves, and increase the chlorophyll content, chlorophyll fluorescence, and Performance Index (PI) in maize plants growing under rain-fed conditions, thereby increasing their drought tolerance [58, 65].

Lu *et al*. [66] studied the role of the *epsC* gene in EPS-producing *B. amyloliquefaciens* FZB42 on drought tolerance mechanisms of Arabidopsis by producing a mutant without *epsC* gene. Inoculation with mutant strains decreased growth and tolerance of plants in drought stress. The results showed that bacterial strain had a significant effect on drought tolerance and growth of the plants as evidenced by increase in survival rate, weight of fresh and dry shoot, weight of dry root, primary and lateral root length, and lateral root number. Additionally, there was also improvement in proline levels, cellular defense responses, and activities of peroxidase and superoxide dismutase. Inoculation with the FZB42 strain was reported to reduce lipid peroxidation and hydrogen peroxide accumulation in plants. Also, there was an increase in the expression levels of marker genes related to drought tolerance such as ERD1, RD17, LEA14, and RD29A in the leaves of plants inoculated with FZB42. The study also supported that the drought tolerance of FZB42-treated Arabidopsis plants was enhanced not by *epsC* *which determines colonization of bacteria in the roots and induces systemic drought tolerance in* Arabidopsis [66]. Plant growth-promoting bacteria isolated from the rhizosphere region of chickpea plant were characterized for the production of EPS, hydrogen cyanide (HCN), indole-3-acetic acid (IAA), and ammonia (NH_3_). It was seen that the protein and sugar content of EPS were 98% and 96% in *Bacillus megaterium*, and 98% and 95% in *B. thuringiensis* and *B. subtilis*. The uronic acid content was found to be maximum (94%) in *B. megaterium*. All these strains were capable of inducing metabolic changes like accumulation of L-arginine, L-isoleucine, proline, L-histidine, tryptophan, L-asparagine, aspartate, riboflavin, nicotinamide, glycerol, and 3-hydroxy-3-methylglutarate in chickpea leaves to induce drought resistance [11, 60]. Biofilm formation by the *B. amyloliquefaciens* 54 strain improved drought resistance by increasing the survival rate, root vigor, and relative water content [67]. This ability of the bacteria was shown to have a significant effect on multiple pathways such as decreasing the concentration of malondialdehyde, increasing the concentration and activity of antioxidant enzymes, and increasing the expression of stress-responsive genes like *ltpg2*, *tdi65*, and lea, as demonstrated by mutant studies of the strain. Plants that were inoculated with mutants capable of hyper-robust biofilm formation (*ΔabrB* and *ΔywcC*) were found to demonstrate drought resistance as compared to plants that were inoculated with mutants having biofilm formation defects (*ΔepsA-O* and *ΔtasA*) which could not grow in water deficit conditions. Therefore, these studies confirm that the ability to form biofilms helped the *B. amyloliquefaciens* 54 strain enhance drought tolerance of tomato plant [67].

Indigenous rhizobacteria capable of forming biofilms such as *Bacillus* species, *Pseudomonas* species, *Brevibacterium* species, *Pantoea* species, and *Acinetobacter* species have demonstrated several significant plant growth-promoting attributes and induced drought tolerance in wheat plants [12, 62]. Similarly, *Arabidopsis thaliana* seedlings inoculated with *Bacillus tequilensis* J12, *Bacillus endophyticus* J13, *P. aeruginosa* ZNP1, and *P. aeruginosa* PM389 strains showed the ability to counteract the adverse effects of osmotic stress conditions by improvement in fresh and dry weight and water content of the plant as compared to uninoculated plants exposed to osmotic stress. Among these four strains, the *B. endophyticus* J13 and *P. aeruginosa* ZNP1 strains demonstrated increased ability for EPS production when exposed to osmotic stress [68].

Different *Rhizobium* species such as *R. strain* R1, *R. tropici* R2, *R. cellulosilyticum* R3, *R. taibaishanense* R4, and *E. meliloti* R5 strains that were isolated from the rhizosphere of Bambara groundnut plants to enhanced seed germination of soybean (PAN 1532 R) plants when exposed to drought. Genomic studies on these strains have revealed the involvement of auxin, *htrA*, *nodA*, *exoX*, *Nif*, *eptA*, and siderophore-producing genes responsible for promotion of plant growth and drought tolerance [69]. Sushilowati *et al*. [70] identified EPS-producing drought-resistant bacteria such as *B. megaterium*, *B. licheniformis*, and *Bacillus* pumilus that imparted drought-tolerant properties to the soybean plant. High EPS formation by ACC deaminase-producing *P. fluorescens* DR7 could colonize the rhizospheric soil and enhanced RAS/RT ratio, supporting that this probably improved the microenvironment of the soil by holding water in drought environment and promoted growth of foxtail millet (*Setaria italica* L.) in arid regions [71].

Another study reported that EPS-producing strains like *P. fluorescens*, *Enterobacter hormaechei*, and *Pseudomonas migulae* isolated from soil of foxtail millet (*S. italica* L.), a drought-tolerant crop, produced EPS in direct relation to the population size of the strain [21]. EPS from *P. chlororaphis* O6 reduced the wilting in *A. thaliana* when applied to seedlings while reducing the stomatal opening when applied to leaf of the plant [72]. Polyssaccharide from *M. arborescens* helps to cement soil particles together and stabilizes the soil [55]. This soil aggregation is dependent on the dose of the inoculum as reported by Vardhajula *et al*., 2014, using *Bacillus* spp. strains HYD-B17, HYTAPB18 and RMPB44 in drought as well as non-stressed conditions [19, 73]. Incubation period also determines the production of EPS and aggregation of soil [73] (Table 1). Microenvironments created by EPS-producing bacteria in soil in drought helps to concentrate minerals in drought stress and support soil structure [49]. These facilitate enhanced nutrient and water uptake through improved root/shoot growth. EPS-producing rhizospheric bacteria increased water efficiency use by 63% by threefold increased soil aggregation around the wheat roots [74]. Similarly, EPS-producing strain *Klebsiella* sp. IG3 led to improved RAS permeability through increasing soil aggregation and water potential around the roots [75]. This discussion supports that plant growth-promoting microorganisms play a significant role in drought tolerance with various mechanisms [76] (Table 2).

## Role of EPS in Mitigation of Salinity Stress in Plants

The problem of increased soil salinity has become a global concern. Some international agencies that have actively participated in data collection regarding soil salinity around the world include the United Nations Educational, Scientific and Cultural Organization – United Nations Environment Programme (UNESCO-UNEP), the Food and Agriculture Organization (FAO), and the International Society of Soil Science (ISSS). According to the Soil Map of the World (FAO, 1971-1981), the problem of high soil salinity affects around 953 million hectares (Mha) worldwide. The FAO report on “Status of the World’s Soil Resources” stated that more than a hundred countries worldwide with a total estimated area of about one billion hectares faces the problem of high soil salinity [77].

High soil salinity mainly occurs due to excessive use of chemical fertilizers and pesticides, lack of proper drainage systems and improper irrigation practices, and this high salt content in the soil turns out to be extremely damaging for crops [78]. Apart from drought, salinity is another common environmental stressor that adversely impacts plant growth and development and decreases crop yields in affected regions globally [79]. The negative effects on plant growth due to salinity can be attributed to osmotic stress, partial stomatal closure, or nutrient imbalance [80]. Some of these negative effects specifically include decreased energy and lipid metabolism, reduced capacity for chlorophyll content, photosynthesis, decrease in total nitrogen content, and reduced protein synthesis [80]. Salinity also causes oxidative stress in plants causing damage to protein, nucleic acid and lipids peroxidation resulting in loss of membrane integrity [81]. Salt stress-tolerant strains produce EPS in varying compositions and concentrations, enhancing germination and improving crop yields under environmental stress conditions [70, 82]. EPS works as a physical barricade in the soil protecting roots and promoting plant growth under high salinity conditions [72, 83]. EPS production by bacteria in high salinity soil enhances its physicochemical properties and promotes soil aggregate formation [83]. Microbes, especially bacteria, have the ability to impart salt-tolerance to plants and enhance their growth through various mechanisms in saline soil [84]. EPS binds with sodium ions thereby reducing the effects of high soil salinity and EPS produced by microbes mitigates salt stress by maintaining Na^+^/K^+^ balance that helps plant to survive under unfavorable soil conditions [85, 86]. EPS-producing salt-tolerant bacteria when inoculated in plants improved the ability of the plants to take up sodium, calcium, and potassium ions from the soil [73, 74]. EPS chelates sodium ions from around the roots thereby preventing the ions from reaching the stem and decreasing sodium absorption from the soil [87]. EPS chelates sodium ions from around the roots thereby preventing the ions from reaching the stem and decreasing sodium absorption from the soil [88]. An increase in salt concentration also stimulates an increase in EPS production which further leads to biofilm production [78, 82, 89] and further enhancement of Na + chelation. NaCl concentration in soil also determines the composition of EPS as increased rhamnose and trehalose were reported by Tewari and Arora [84]. These sugars help microbes to tolerate salt stress by providing carbon source, enhancing water retention.

Researchers have shown that both biofilm formation and EPS production result in several advantages to plants growing in a high salinity environment. They increase cell viability in the rhizosphere region thereby enhancing soil fertility and plant growth [88]. They protect plants from external stress, increase surface area for adhesion, provide high population densities, enhance plant tolerance to antimicrobial agents, and promote nutritional competition between microorganisms [90]. Various strategies adopted by microbes to alleviate salt stress include osmotic balancing, ion transport, and activating oxidative stress defense mechanisms [12, 90-92]. Also, microbes produce various phytohormones, ACC deaminase, siderophores and exopolysaccharides [92]. Studies using the halotolerant strain *R. meliloti* EFB1 showed that two EPSs, a succinoglycan (EPS I) and a galactoglucan (EPS II) are produced and regulated at the transcriptional level in response to soil salinity [93].

Upadhyay *et al*. [12] demonstrated that PGP traits such as phosphate solubilization, auxin production, sugar reduction, proline production, and total soluble sugar production were shown by EPS-producing salt-tolerant bacterial strains *Bacillus* sp. (SKU-3) and *Paenibacillus* sp. (SKU 11) which helped in increasing the plant biomass in inoculated plants when compared to uninoculated plants [12]. Also, under conditions of high salinity, greater bacterial enrichment was noticed in the rhizosphere region when compared to normal salinity conditions. All these strains belonged to the genera *Bacillus*, *Burkholderia*, *Enterobacter*, *Microbacterium*, and *Paenibacillus* and showed EPS and IAA production capability, which decreased with increase in soil salinity [12, 68].

Qurashi and Sabri [82] demonstrated that the EPS-producing salt-tolerant plant growth-promoting rhizosphere strains, *Planococcus rifietoensis* RT4 and *Halomonas variabilis* HT1, could stabilize soil structure and promote soil aggregation under high salinity conditions, which had a positive effect on growth of chickpea plant. EPS production by bacteria in the biofilm also helped in root colonization by salt-tolerant plant growth-promoting rhizhosheric bacteria. The two strains *P. rifietoensis* RT4 and *H. variabilis* HT1 produced increased EPS and biofilm formation under high salinity conditions which supported the growth of chickpea plant [82]. Atouie *et al*. [94] studied EPS-producing strains *B. subtilis* subsp. inaquosorum and Marinobacter lipolyticus SM19 (T) on increasing plant resistance to salt and drought stresses by decreasing Na^+^ uptake and increasing the dry weight of root in wheat.

In a recent study, EPS-producing *Pseudomonas* PS01 strain upregulated the lipoxygenase (LOX2) gene in *A. thaliana*, which codes for a lipoxygenase that plays an important role in the jasmonic acid (JA) synthesis pathway. JA is known to accumulate in plants under salt stress conditions and act as a positive regulator of stress-responsive genes [95, 96]. Additionally, EPS production by bacteria helps plants survive under high salinity conditions. The mutant that was produced by random transposon mutagenesis was used to study the changes responsible for stress tolerance, which included decreased EPS production, low tolerance to salinity, and reduced competitive fitness in the rhizosphere [96]. Salt-tolerant bacterial isolates such as *Achromobacter denitirificans*, *Bacillus aryabhattai*, and *Ochrobactrum intermedium* demonstrated increased ability to fix atmospheric nitrogen and increase phosphate solubilization and IAA production when exposed to salt stress of 200 mmol/L. Based on their ability to produce EPS, these isolates demonstrated higher resistance to antibiotics and heavy metals along with increased expression of salt-responsive genes in the plant such as SOS1, NHX1, GIG, and BZ8. Therefore, inoculation of the rice plant with salt-tolerant plant growth-promoting bacteria can be a potentially good strategy for coastal agriculture [97]. Further exploration showed EPS with high glucose could chelate Na + present in saline soil, preventing its availability to the plant. Inoculation of *B. tequilensis* and *B. aryabhattai* strains increased photosynthesis, transpiration, and stomatal conductance of the plant leading to high yield under saline stress in rice crop [17].

*Mesorhizobium alhagi* is a soil bacterium that forms a symbiotic relationship with legumes, specifically forming nodules with the desert plant Alhagi sparsifolia, as revealed by the results of Mu *et al*. phenotypic analysis. It uses mannitol as a carbon source and produces a large quantity of EPS. EPS-deficient mutants of *M. alhagi*, CCNWXJ12-2^T^, which was constructed using transposon mutagenesis, demonstrated decreased tolerance to salt stress, low antioxidant capacity, and reduced cell motility when compared to the wild-type strain. This also proves that EPS plays a role in maintaining cellular sodium content and activity of antioxidant enzymes to optimum levels, which helps in adapting to high salinity [98]. EPS-producing bacteria (*Azotobacter chroococcum*) and melatonin alleviated salinity stress on faba bean plants by decreasing Cl^−^ concentrations. Also, bacterial priming and meltonin enhanced N, P, and K concentrations; the proline content; relative water content RWC%; and the K^+^/Na^+^ ratio [99]. Salt tolerance in *Rhodopseudomonas palustris* strains TN114 and PP803 is attributed to galacturonic acid, a polysaccharide (≈18 kDa) present in EPS, chelates Na^+^ cations from aqueous environment [100]. Similarly, the salt-tolerant plant growth-promoting R strains, *Bacillus* sp. (PM15), *B. siamensis* PM13, and *B. methylotrophicus* PM19 have demonstrated the ability to attenuate the adverse effects of high soil salinity in wheat plant [101,91]. Several bacterial genera, *Pseudomonas*, *Bacillus*, *Burkholderia*, *Enterobacter*, *Microbacterium*, *Planococcus*, *Halomonas*, and *Azotobacter* have been reported to produce EPS in salt stress condition [82, 102]. Also, they support plant growth by inducing indole acetic acid production, biological nitrogen fixation, solubilization of soil P and K, and production of siderophores and hydrolyzing enzymes under salt stress condition [12]. Mohammad *et al*. [103] showed high PGP and biofilm-forming activity of *Pseudomonas anguilliseptica* SAW 24 and hence correlation between these traits under saline and non-saline conditions. Similarly, combined application of ACC deaminase and EPS-producing *Bacillus* isolates and *Rhizobium* improved faba beans seedling growth under saline conditions by accumulation of sugars and proteins [104]. *Enterobacter* sp. MN17 and *Bacillus* sp. MN54 improved plant-water relations in quinoa (*Chenopodium quinoa*) under saline stress [105]. *Rhizobium* and *Pseudomonas* co-inoculation in maize was reported to increase proline production and decreased electrolyte leakage along with maintenance of leaf relative water content, and uptake of K^+^, as a mechanism in imparting salt tolerance [106]. *Aeromonas hydrophila/caviae* MAS765, *Bacillus insolitus* MAS17, and *Bacillus* sp. strains MAS617, MAS620, and MAS820 restricted passive flow of Na^+^ from the layer of soil clinging roots to stele in wheat crop [107]. A consortium of salt-tolerant Aeromonas spp. SAL-17 and SAL-21 produced Acyl homoserine lactones (AHLs), QS molecules, and sustained the growth of salt-tolerant and non-salt tolerant wheat genotypes in saline soil [108].

This discussion concludes that bacterial exopolysaccharides ameliorate salinity stress by chelating free Na^+^ ion, making it available to plants, supporting soil aggregation and stability, enhancing biofilm formation, and contributing to water retention (Table 3). There are several reports on protection of plants in salinity by EPS-producing plant growth-promoting microorganisms, still, more studies are needed about change in physicochemical properties of EPS in saline environment.

## Role of EPS in Mitigation of Metal Stress in Plants

Soil gets contaminated with heavy metals due to natural phenomena such as soil erosion, volcanic eruptions, weathering of minerals, and forest fires, or by anthropogenic activities such as excessive use of pesticides and chemical fertilizers, smelting, mining, automobile exhaust emissions, leather tanning, municipal waste disposal, textiles dyeing and processing, and manufacturing industrial activities [109]. Heavy metals are toxic in nature because they are non-biodegradable, mutagenic, carcinogenic, and teratogenic, have low bioavailability, and are highly soluble in aqueous environments [110]. The aqueous layer of the soil is a dynamic environment where chemical reactions, and circulation and transfer of heavy metals between bacteria, aqueous layer, and soil layer take place constantly [111]. Heavy metal stress has detrimental effects on plant growth by decreasing chlorophyll contents, blocking gas exchange factors and production of ROS, all of which impose oxidative stress [112].

Plant-associated microorganisms produce EPS and biosurfactants which help in maintaining soil structure and soil fertility in adverse toxic metal conditions [113]. Another study by Mishra *et al*. [112] reported that polysaccharides and lipopolysaccharides of EPS remove metal from rhizospheric soil by biosorption of metal ions owing to presence of anionic functional group. These groups include phosphate, hydroxyl, succinyl, and uronic acids. ESP can mobilize toxic metals by binding with heavy metals such as cadmium, lead, zinc, uranium, copper, and iron aluminum [112].

EPS affinity to heavy metals is due to electrostatic interactions which occur between heavy metal ions and surface functional groups of EPS such as hydroxyl and carboxyl. It binds with cationic heavy metals such as Cd^2+^, Co^2+^, Pb^2+^ and Ni^2+^ resulting in the formation of EPS-metal complexes [16]. Trivalent cations compete with divalent cations to bind with EPS, and trivalent cations form stronger bonds with EPS [114]. Cr (VI)-tolerant plant growth-promoting strain *Cellulosimicrobium funkei* KM032184 isolated from *Phaseolus vulgaris* L. showed tolerance to multiple metal and plant growth-promoting traits. Metal tolerance was due to entrapping of metal ions by the anionic molecules of EPS which restricts the mobility of metal ions to plants [115]. EPS- and ACC-producing *Bacillus gibsonii* PM11 and *Bacillus xiamenensis* PM14, isolated from sugarcanés rhizosphere, promoted growth of flax plant (*Linum usitatissimum* L.) under multi-metal contaminated soil through elevated phytoextraction of metals [116].

Cyanobacterial EPS helps in preventing direct contact between these toxic metals and plant cells [117]. Jittaquttipoka *et al*. [117] demonstrated four genes namely, *sll0923*, *sll1581*, *slr1875*, and *sll5052*, that are responsible for secretion of EPS which provide tolerance to *Cynobacterium synechocystis* PCC6803 against NaCl, CoCl_2_, CdSO_4_, and iron starvation.

Many bacterial strains like *Pseudomonas* and *Enterobacter* play an important role in bioremediation of heavy metals. A study demonstrated the lead bioremediation properties of *Pseudomonas* sp. W6 isolated from hot water springs in Northeast India. This *Pseudomonas* strain showed a high capacity for adherence to metal substrates and soluble metals [118].

Gutehan *et al*. [119] isolated 66 EPS-producing plant growth-promoting bacteria with variable tolerance to Fe, Mn, Cu, Zn, and Pb, Fe and Mn which promoted growth of *Acacia* in metal stress. Out of these, *Paenibacillus polymyxa* strain FB50 *Acinetobacter calcoaceticus* stain BS-27, *P. putida* strain BS-19, and *P. fluorescens* strain FB-49 showed 100% tolerance to all heavy metals. *Paenibacillus* spp and *B. thuringiensis* were also tolerant to heavy metals like Cd, Cu, and Zn. Similarly, two plant growth-promoting rhizospheric bacterial strains, *B. gibsonii* PM11 and *B. xiamenensis* PM14, isolated from sugarcanés rhizosphere, showed tolerance to multiple metals (Cd, Cr, Cu, Mn, and Zn) [116]. These are also reported to possess plant growth-promoting attributes like IAA, ACC-deaminase, EPS production and nitrogen fixing, and siderophore production under metal stress. Flax plant (*L. usitatissimum* L.) could survive and showed improved growth in metal stress when primed with *B. gibsonii* PM11 and *B. xiamenensis* PM14 [116].

The *Halomonas* species isolated from the rhizosphere of the true mangrove *Avicennia marina* secrete EPS which sequestered arsenic and salt in both in vitro and in vivo studies, proving EPS has a direct role in plant growth promotion in metal stress environment [120]. EPS-producing, cadmium- and chromium-tolerant strain *Bacillus* *anthracis* PM21 enhanced seed germination, root and shoot length and photosynthetic pigment in *Sesabania sesban* in metal-stressed conditions. Inoculation with this strain also increased activities of antioxidant enzymes superoxide dismutase, peroxidase, and catalase, and decreased proline content, electrolyte leakage and malondialdehyde concentration in seedlings [121] (Table 4). This discussion shows plant growth-promoting microorganisms with EPS production have significant ability for heavy metal bioremediation. Engineering or modifying such microbes for EPS production to enhance heavy metal removal can be useful for the growth of plants in metal stress environments [122, 123].

## Role of EPS in Mitigation of Temperature Stress in Plants

High temperature-induced heat stress causes damage to crop plants, [124] subjecting global crop production to great peril [125]. Global warming could cause an increase in global temperature from 1.6 to 6°C by 2050 FAO [46]. A rise by one degree in temperature will lead to 20% decrease in water resources and this is a major concern for agricultural productivity [126]. Climate change affects plant metabolism, seed germination, and seedling growth, photosynthesis, chloroplast metabolism thereby reducing crop yields [127]. At high temperatures (30-38°C), delay in seed germination can be observed [120]. The reproductive stage of a plant is especially sensitive to high temperatures, and this has been demonstrated in chickpea [128], mungbean [129], sorghum [130], wheat [131], and lentil [132] plants. In tropical and temperate regions, flowering, number of flowers, and number of fruits in a plant are also affected by heat stress. Plant growth-promoting rhizosphere bacteria such as *Pseudomonas* produce EPS which serve as a defense mechanism for microorganisms to survive under stress conditions [133]. Therefore, this activity of such microorganisms helps attenuate the adverse effects of high temperatures on plant growth, development, and crop productivity [134]. Mukhtar *et al*. [135] showed an increase in the number of fruits and flowers in two varieties of tomato plant, *i.e.*, Sweetie and Riogrande, upon inoculation with EPS-producing *B. cereus*, in high temperature stress. Plant growth-promoting microbes inoculation was found to improve physiological traits such as length of root and shoot, dry weight and fresh weight, and surface area of leaves, and biochemical traits such as chlorophyll content, relative water content, protein content, proline content, and antioxidant enzyme activities in the tomato plant when exposed to high temperature. Environmental heat stress induced changes in EPS production and production of new types of cellular proteins such as heat shock proteins. Heat shock proteins prevent aggregation, assist in protein refolding, and target abnormally folded proteins for degradation [136]. Nandal *et al*. [137] demonstrated the adaptive mechanism of *Rhizobia* species under heat stress conditions. At 43°C, EPS production was higher in all 14 heat-resistant mutant strains as compared to the parent strain (PP201).

Nguyena *et al*. [138] demonstrated EPS-producing *Bfidobacterium bifidum* survived when exposed to a sub-lethally high temperature. Increased cell robustness, cell viability, and stress resistance, and a reduction in the net surface charge of microbial cells and survival against freeze-drying was observed. *Pseudomonas* sp. strain PsJN promoted growth in vitro and ex vitro, of various varieties of potato under heat stress conditions [139]. Al-Abd Daim *et al*. [140] evaluated management of heat stress in two wheat varieties (Olivin and Sids1) under heat stress conditions and revealed that the application of *Bacillus amyloliquefaciens* UCMB5113 or *A. brasilense* NO40 improved the growth of plants in heat stress. Later, in another set of experiments, Al-Abd Daim *et al*. [140] reported plant growth-promoting rhizospheric strain *Bacillus velezensis* 5113 inoculation resulted in a metabolic modulation and involvement of many regulatory proteins in wheat leaves to develop heat, cold and drought stress tolerance.

Another investigation on the role of two bacterial strains, *B. aryabhattai* H26-2 and *B. siamensis* H30-3 on mitigation of heat and drought stress on two cultivars ('Ryeokkwang' and 'Buram-3-hó) of Chinese cabbage suggested exopolysaccharides as important bacterial determinants on alleviating not only these stresses but also on biocontrol activity against soft rot caused by *Pectobacterium carotovorum* subsp. *carotovorum* PCC21[141].

Heat stress (45oC) affected knockout mutants *Shinorizobium meliloti* HslUV and ClpXP proteases and reduced EPS production and shoot dry weight in *Medicago sativa* suggesting the role of EPS in symbiosis during heat stress [142]. Similarly, *Bradyrhizobium diazoefficiens* USDA110, responsible for nitrogen fixation in soybean, accumulates poly-3-hydroxybutyrate (PHB), a source of carbon/energy during starvation, infection, and nitrogen fixation conditions. PHB synthesis involves pha gene regulated by PhaR transcription factor. Inactivation of this factor decreases PHB accumulation and cell yield but enables accumulation of EPS and heat stress tolerance. This suggests a direct role of EPS in heat stress tolerance [142, 143] (Table 5).

There are still many mechanisms needing to be explored for abiotic stress responses using plant growth-promoting rhizospheric bacteria and EPS and applied plant growth-promoting microorganism (PGPM) bacterial strains shown to improve crops growing under heat-stressed conditions.

Like high temperature stress, plant productivity is also deteriorated under cold stress. Psychrophiles and psychrotolerant microorganisms colonize permanently cold habitats, such as the polar regions, high altitudes and the deep sea and grow at temperatures ranging from subzero to 15oC. At high-altitude, psychrotolerant microbes sustain and maintain functionality in cold-temperature conditions, while growing optimally at warmer temperatures. Low temperature primarily causes irreversible freezing injury which includes dysfunction of plasma membrane and cellular dehydration [144]. Plant growth-promoting bacteria present within the root zone stimulate the increase in density of root hairs through various phytohormones and hence increase the uptake of water and nutrients. There are investigations reporting on the role of such microorganisms in protecting crops grown at low temperature. Psychrophilic *Bacillus* strains CJCL2 and RJGP41 were able to significantly improve wheat growth positively by regulating abscisic acid, lipid peroxidation and proline accumulation pathways [145]. In other studies, *Burkholderia phytofirmans* PsJN could tolerate cold stress by inducing high concentration of total reducing sugars, glucose, and proline on bacterization of grapevine [146, 147]. A consortium of strains (*B. cereus* AR156, *B. subtilis* SM21, and *Serratia* sp. XY21 also increased accumulation of soluble sugar, proline, and osmotin, enhanced the antioxidant defense system, and activated stress-related genes in tomato at chilling shock of 4oC [148]. Bacterization of wheat with psychrotolerant *Pseudomonas* at cold temperature alleviated cold stress by improving chlorophyll, anthocyanin, free proline, total phenolics, starch content, physiologically available iron, proteins, and amino acids. Other physiological traits like increased EPS, relative water content, reduced membrane injury (electrolyte leakage), and Na^+^/K^+^ ratio also improved [144]. However, few studies are available addressing the exact role of EPS-induced protection under cold/chilling stress on plants.

The above discussion supports that EPS-producing microorganisms are key players in sustaining and increasing the productivity of the existing agro-ecosystem through myriad roles under varied abiotic stress conditions (Fig. 3 Graphical Abstract).

## Conclusion and Future Perspective

Agricultural productivity and crop yields can be affected by various environmental stressors such as drought, salinity, high temperatures, and heavy metals, all of which adversely affect plant growth and development, and eventually lead to global food scarcity. Recent investigations have identified several species of bacteria that impart stress tolerance properties to plants through various activities such as EPS production and biofilm formation, which help increase the nutrient uptake and water retention capacity of plants. These PGP bacteria have been extensively studied as bio-inoculants used to promote plant growth and seed germination in regions of heavy environmental stress. They have been demonstrated to possess several PGP traits such as the production of siderophores, IAA, phytohormones and EPS that make plants resistant to environmental stress. Although the review of literature has shown promising results in this area of research, further studies and field trials are needed to characterize the PGP attributes of EPS produced by microorganisms under different stress conditions that are beneficial to plant growth and metabolism. Also, elaborate research is needed on the reciprocal effect of abiotic stress and EPS composition and the physicochemical nature of EPS under such stress conditions. These studies would help to provide tools for alleviating abiotic stress in agricultural crops. The combined use of EPS-producing bacteria offers a promising strategy for multiple abiotic stress management for crop plants. Additionally, bacterial EPS is appropriate for maintaining soil composition, nutrients and fertility to improve plant growth and crop yields.

## Figures and Tables

**Table 1:** EPS-producing bacterial strains in soil aggregation.

Microorganisms	Roles of EPS	References
*Microbacterium arborescens*	Cementing of soil particles	[55]
*Bacillus* sp.	Soil aggregation	[88]
*Pseudomonas mendocina*	Promote soil fertility and stabilization	[51]
*Rhizobium* sp.	Soil aggregation Increase in water holding capacity of soil in rhizosphere	[48]
*Pseudomonas putida* strain GAP-p45	Soil aggregation increase root adherence stability Biofilm formation on surface of root of sunflower seedling	[52]

**Table 2:** EPS-producing microbial strains in drought tolerance.

Microorganisms	Crops	Functions	References
*Rhizobium* sp. strain YAS34	Sunflower	Increase RAS/RT ratio, dry biomass, and nitrogen uptake	[24]
*Pseudomonas mendocina*	Lactuca sativa L	Increase in aggregate stability, water soluble, and total carbohydrates	[50]
*Proteus penneri* *Pseudomonas aeruginosa* *Alcaligenes faccalis*	Maize	Improve soil moisture content, leaf area, root length, shoot length, and plant biomass	[56]
*Bacillus* sp. (*B. amyloliquefaciens*, *B. licheniformis*, *B. thuringiensis*, *B. subtilis*, *Paenibacillus favisporus*)	Maize	Increase plant biomass, relative water content, water potential of leaf, RAS/RT ratio, and aggregate stability	[11]
*Rhizobium leguminosarum* *Mesorhizobium ciceri* *Rizobium phaseoli*	Wheat	Improve growth, biomass, and drought tolerance index	[58]
*Rhizobium* strain KYGT207	Wheat	Improve soil aggregation	[48]
*Bacillus* sp. (*B. licheniformis*, *B. megaterium*, *B. pumilus*)	Soybean	Plant growth promotion	[70]
*Klebsiella* sp. IG3	Wheat	Improve soil aggregation, root adhering soil, and water potential around the roots	[75]
*P. aeruginosa* PM389 *P. aeruginosa* ZNP1 *B.endophyticus* J13 *B. tequilensis* J12		Increase phytohormones/EPS Enhance fresh weight, dry weight, and plant water content	[68]
*Pseudomonas chlororaphis* O6	*Arabidopsi s thaliana*	Reduce stomatal opening and wilting	[72]
*B. amyloliquefaciens* 54	Tomato	Increase survival rate, relative water content, and root vigor Decrease concentration of malondialdehyde Increase antioxidant enzyme activities Increase levels of stress-responsive genes (*lea*, *tdi65*, and *ltpg2*)	[96]
*B. amyloliquefaciens* FZB42	*A. thaliana*	Increase survival rate, fresh and dry shoot weights, and primary root length Increase expression levels of drought defense-related marker genes, such as *RD29A, RD17, ERD1*, and *LEA14* Increase proline production and activities of superoxide dismutase and peroxidase	[66]
*Pseudomonas fluorescens* DR7 *P. fluorescens* DR11 *Pseudomonas migulae* DR35 *Enterobacter hormaechei* DR16	Foxtail millet	Increase the RAS/RT Improve seed germination	[71]
*P. fluorescens* FAP2 *B. licheniformis* B642	Wheat	Enhance vegetative growth Enhance photosynthesis, chlorophyll content, transpiration rate (E), internal CO_2_ concentration (Ci), stomatal conductance (gs), net photosynthetic rate (PN), and leaf water potential (LWP) as compared to uninoculated control	[62]

**Table 3:** Applications of EPS-producing salt-tolerant bacteria.

Microorganisms	Crops	PGP trait	References
*Halmonas variabilis HT1* *Planococcus refietoensis* RT4	*Cicera rietinum*	Increase in fresh weight, dry weight, total soluble sugars and protein contents Increase soil aggregation and biofilm formation	[82]
*Rhizobium meliloti*	Tomato	EPS with high succinoglycan and galactoglucon	[67]
*Bacillus* sp., (*B. licheniformis*, *B. pumilus*, *B. coagulans*, and *B. insolitus*) *Paenibacillus* sp., (*P. macerans*) *Microbacterium* sp. *Burkholderia cepacia* *Enterobacter* sp.	Wheat	Rhizospheric soil aggregation Decrease Na^+^ uptake and root biofilm formation	[12]
*Pseudomonas anguilliseptica* SAW 24	Faba bean	Enhance plant height and fresh/dry weight	[81]
*Rhizobium* and *Pseudomonas*	*Zea Mays*	Increase proline production Decrease electrolyte leakage along with maintenance of leaf relative water content and uptake of K^+^	[106]
*Bacillus isolates* and *Mesorhizobium*	*Cicer arietinum* L. (Chickpea)	Reduce concentration of Na^+^ in soil	[104]
*Azotobacter chroococcum*	*Vicia faba* L.	Enhanced N, P, and K concentrations, the proline content, RWC%, and the K^+^/Na^+^ ratio	[99]
*Aeromonas* spp. SAL-17 and SAL-21	Wheat	Increase in leaf proline content, nitrate reductase activity, chlorophyll a/b, stomatal conductance, transpiration rate, root length, and shoot length	[108]
*Aeromonas hydrophila/caviae* MAS765, *Bacillus insolitus* MAS17, and *Bacillus* sp. MAS617, MAS620, and MAS820	Wheat	Restricted passive flow of Na^+^ from the roots to stele in wheat crop and large root surface covered with soil	[107]
*Marinobacter lipolyticus* SM19 and *B. subtilis* subsp. *inaquosorum*	Wheat	Shoot and root dry weight Restricted Na^+^ uptake	[94] [83]
*Pseudomonas aeruginosa* PF07	*Hellianthus annuus*	Enhance Root Adhering Soil to Root Tissue ratio (RAS/RT)) and texture of the soil Increase porosity Improve uptake of nutrients	[84]
*Enterobacter* sp. MN17 and *Bacillus* sp. MN54	Quinoa (*Chenopodium quinoa*)	Improved plant-water relations	[105]
*Rhodopseudomonas palustris* TN114 and PP803	-	Chelate Na +cations from aqueous environment	[100]

**Table 4:** EPS-producing bacteria demonstrating metal tolerance.

Microbial Strain	Metals	Plants	Roles of EPS	References
*Cynobacterium synechocystis* PCC6803	Cadmium, Cobalt	-	Tolerance to stresses triggered by NaCl, CdSO_4_, CoCl_2_, or Fe starvation	[117]
*Pseudomonas* sp. W6	Lead	-	Bio-adsorption of lead	[118]
*A. calcoaceticus* BS-27 *P. polymyxa* FB-50 *P. putida* BS-19 *P. fluorescens* FB-49	Iron, Manganese, lead, Zinc, Copper	Acacia	Increase root and shoot length, dry biomass, and metal tolerance	[119]
*Bacillus gibsonii* PM11 *Bacillus xiamenensis* PM14	Iron, Manganese, Cadmium, Zinc, Copper, Chromium	Flax (*Linum usitatissimum* L.)	Enhance plant growth and nutrient availability by minimizing metalinduced stress Enhance phytoextraction of multimetals	[116]
*Bacillus anthracis* PM21	Cadmium, Chromium	*Sesbania sesban*	Enhance seed germination, root and shoot length and photosynthetic pigment	[121]
*Cellulosimicrobium funkei* KM032184	Chromium	*Phaseolus vulgaris* L	Root Elongation, Shoot elongation, Antioxidant,	[115]
*Halomonas* species	Arsenic	*Avicennia marina*	Arsenic bioadsprtion, in vitro Na^+^ ion sequestration and antioxidant activity	[120]

**Table 5:** EPS-producing PGPR imparting tolerance to heat stress.

Microorganisms	Crops	Roles of EPS	References
*Bacillus cereus*	Tomato	Increase the number of flowers and fruits Increase chlorophyll, proline and antioxidants	[135]
*Bacillus amyloliquefaciens* UCMB5113 *Azospirillum brasilense* NO40	Wheat	Increase HSP26 and chlorophyll content Accumulate GABA and modulate metabolic pathways	[140]
*Pseudomonas* sp. AKM-P6	Sorghum	Enhance tolerance of sorghum seedlings to elevated temperatures	[134]
*Rhizobium* sp. (*Cajanus*)	Legume	Heat shock protein (Hsp) of 63-74 kDa	[29]
*Pseudomonas* sp. PsJN	Potato	Promote growth	[139]
*Bacillus aryabhattai* H26-2 and *Bacillus* siamensis H30-3	Chinese cabbage	Leaf abscisic acid (ABA) content and reduced stomatal opening after stresses treatments, Biocontrol activity against soft rot	[140]
*Shinorizobium meliloti*	*Medicago sativa*	Affect symbiosis during heat stress	[142]
*Bradyrhizobium diazoefficiens* USDA110	Soybean	Survival in starvation	[143]
